# Assessing long‐term diatom changes in sub‐Arctic ponds receiving high fluxes of seabird nutrients

**DOI:** 10.1002/ece3.11034

**Published:** 2024-02-16

**Authors:** Kathryn E. Hargan, Matthew P. Duda, Neal Michelutti, Jules M. Blais, John P. Smol

**Affiliations:** ^1^ Department of Biology University of Ottawa Ottawa Ontario Canada; ^2^ Paleoecological Environmental Assessment and Research Laboratory, Department of Biology Queen's University Kingston Ontario Canada

**Keywords:** Arctic, diatoms, environmental change, freshwater ponds, paleolimnology, primary production, seaducks

## Abstract

Algal bioindicators, such as diatoms, often show subdued responses to eutrophication in Arctic lakes because climate‐related changes (e.g., ice cover) tend to be the overriding factors influencing assemblage composition. Here, we examined how sub‐Arctic ponds historically receiving high nutrient inputs from nesting seabirds have responded to recent climate change. We present diatom data obtained from 12 sediment cores in seaduck‐affected ponds located on islands through Hudson Strait, Canada. All study cores show consistently elevated values of sedimentary ẟ^15^N, an established proxy for tracking marine‐derived nutrients, indicating seabirds have been present on these islands for at least the duration of the sediment records (~100 to 400 years). We document diverse epiphytic diatom assemblages to the base of all sediment cores, which is in marked contrast to seabird‐free Arctic ponds—these oligotrophic sites typically record epilithic diatom flora prior to recent warming. Diatoms are likely responding indirectly to seabird nutrients via habitat as nutrients promote the growth of mosses supporting epiphytic diatom communities. This masks the typical diatom response to increased warming in the Arctic, which also results in habitat changes and the growth of mosses around the pond edges. Changes in sedimentary chlorophyll *a* were not consistently synchronous with large changes in ẟ^15^N values, suggesting that primary production in ponds is not responding linearly to changes in seabird‐derived nitrogen. Across all ponds, we recorded shifts in diatom epiphytic assemblages (e.g., increases in % relative abundance of many *Nitzschia* species) that often align with increases in chlorophyll *a*. The changes in diatoms and chlorophyll *a*, although variable, are most likely driven by climate change as they are generally consistent with longer ice‐free conditions and growing seasons. Together, our results show that to effectively use diatoms in animal population reconstructions across the sub‐Arctic and Arctic, a strong understanding of eutrophication and climate change, based on supplementary proxies, is also required.

## Graphical abstract

We document diverse epiphytic diatom assemblages to the base of all sediment cores, which is in marked contrast to seabird‐free Arctic ponds—these oligotrophic sites typically record epilithic diatom flora prior to recent warming. Diatoms are likely responding indirectly to seabird nutrients via habitat as nutrients promote the growth of mosses supporting epiphytic diatom communities. Changes in sedimentary chlorophyll *a* were not consistently synchronous with large changes in ẟ^15^N values, suggesting that primary production in ponds is not responding linearly to changes in seabird‐derived nitrogen.

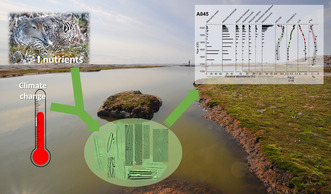

## 1 INTRODUCTION

With climate change, the expansion of aquatic habitats in Arctic ponds has led to a major reorganization of algal communities (Rühland et al., 2015; Smol et al., 2005). In particular, this change in algal community has been widely captured and described through the transition from very simple diatom (Class: Bacillariophyceae) assemblages consisting of a few small, benthic taxa toward a more complex, species‐rich assemblage consisting of epiphytic taxa (Griffiths et al., 2017; Smol, 2023). These epiphytic taxa are associated with littoral habitats and mossy substrates that frequently appear with warming‐induced habitat expansion as ice cover diminishes (Griffiths et al., 2017; Rühland et al., 2015). Diatoms respond to a wide range of environmental cues, and often to both physical and chemical triggers simultaneously (e.g., stratification and lower nutrients). Understandably, climatic signals are tracked more clearly by diatoms in waterbodies that have experienced a minimal degree of previous disturbances, thus having minimal confounding effects to disentangle, as opposed to waterbodies influenced by multiple environmental stressors (Smol, 2010).

Diatoms are widely used as biomonitoring tools to track the direct and indirect limnological impacts of nutrient pollution (Bennion et al., 2010; Pillsbury et al., 2019). In temperate regions, relatively small changes in nutrient inputs to a lake often result in dramatic shifts in diatom assemblage composition, typically among planktonic diatom species (Hall & Smol, 2010; Rühland et al., 2010). This has resulted in numerous diatom‐based models for reconstructing historical lake phosphorus concentrations (Cumming et al., 2015; Enache & Prairie, 2002; Hall & Smol, 1992). However, in many Arctic waterbodies, eutrophication does not trigger the same response in diatom assemblages; rather, increases in epiphytic taxa and more diverse, complex assemblages are often observed (Keatley et al., 2011; Michelutti, McCleary, Douglas, & Smol, 2013). In Arctic waterbodies free of nutrient subsidies (e.g., no seabirds nesting in a waterbody's catchment, no sewage entering the waterbody), limnological changes are more closely linked to climate factors, notably warming reducing ice cover and thus allowing expansion of mosses and aquatic plants along pond shorelines, increasing epiphytic diatom taxa presence relative to epilithic diatom taxa (Smol, 2023; Smol et al., 2005). In regions of the Arctic that have been historically warmer or received high nutrient inputs, epiphytic diatom taxa are usually present throughout the pond's history and the response among these taxa to climate change may be more difficult to observe than it would be in seabird‐free ponds. For example, recent diatom research at historically warmer High Arctic oasis sites recorded a muted diatom response to warming compared with traditionally cold regions because epiphytic habitats have always been available within the warm oasis ponds (Griffiths et al., 2017). Thus, in Arctic regions, climate is often an overriding factor structuring diatom assemblages with strong moderating controls on all fundamental aquatic processes, including ice cover, length of the growing season, habitat alterations and nutrient availability, such that diatom species composition, and abundance can be linked to regional microclimate patterns (Griffiths et al., 2017; Keatley et al., 2008; Smol, 2023; Smol et al., 2005). Arctic ponds that receive substantial fluxes of nutrients may show delayed or subdued responses to eutrophication, and the additional nutrients may mute the algal response to climate change as in shallow ponds it will often be between epiphytic taxa as opposed to a shift between epilithic and epiphytic taxa. However, it is unclear how Arctic lakes and ponds receiving high nutrient loads over many centuries have responded to anthropogenic climate change.

Seabirds, in particular, are potent biovectors that introduce significant nutrient subsidies to the environment, structuring both terrestrial (Duda, Glew, et al., 2020; Mallory et al., 2015; Zwolicki et al., 2013) and aquatic ecosystems (Blais et al., 2005; Duda et al., 2018; Duda, Michelutti, et al., 2021; Keatley et al., 2009). The waste products from large seabird colonies (e.g., feces, molted feathers and eggshells) enrich nearby freshwater ecosystems with nutrients and leave distinct biogeochemical signatures in the sediments (Duda, Hargan, et al., 2021; Michelutti, Blais, Mallory, et al., 2010). From a conservation perspective, there continues to be growing interest in using bioindicators and paleolimnological approaches to reconstruct long‐term population dynamics, which allows for recent seabird declines to be viewed within the context of natural variability (Birks, 2012; Duda, Hargan, et al., 2021; Froyd & Willis, 2008). To improve the use of diatoms in seabird reconstructions across the sub‐Arctic and Arctic, a better understanding of eutrophication responses in Arctic waterbodies is required.

Diatoms are foremost among the bioindicators used to assess changing water quality as they are taxonomically identifiable and their remains are well‐preserved in high abundances in most aquatic environments (Battarbee et al., 2001; Hall & Smol, 2010). Although diatoms have been used to successfully track changes in seabird colony size in maritime (Duda, Allen‐Mahé, et al., 2020; Duda, Robertson, et al., 2020) and temperate (Stewart et al., 2015) regions, as well as nutrient loading to lakes from migratory salmon in British Columbia, Canada (Chen et al., 2011; Gregory‐Eaves et al., 2003); they appear to have limited sensitivity to marine‐derived nutrient loading in Arctic ponds (Hargan et al., 2017; Keatley et al., 2011). Previously, the reduced response of diatom assemblages in Arctic environments to consistently track changes in δ^15^N values, a proxy for marine‐derived nutrients, suggests that diatoms should not be used in isolation as a proxy for changes in trophic status in polar eutrophic sites (Keatley et al., 2011).

In this study, we expanded on previous foundational work by examining the response of diatoms to climate change in historically eutrophic (from avian enrichment) Arctic ponds while further accessing the plausibility of using diatoms as reliable bioindicators to track seabird colonies through time in Arctic waterbodies. We employed a multiproxy approach to reveal the main drivers of diatom assemblage changes across 12 ponds receiving seabird‐mediated nutrient loading. Namely, we used stable nitrogen isotopes (ẟ^15^N) to directly track seabird inputs to ponds (significantly correlated to fecal lipids from seabirds in Hargan et al., 2019), sedimentary chlorophyll *a* to track pond production, and C:N ratios to track changes in the primary organic matter sources to the ponds. Previous research on the same sediment cores showed that seabirds have been present on these Arctic islands for the time captured in the records (Hargan et al., 2019), and thus, we were able to examine the diatom assemblage response to climate warming while ponds have persistently received excess nutrient fluxes over the 19th to 21st centuries.

Our objectives were to (1) determine the main drivers of diatom assemblage changes in Arctic ponds with historically high nutrient loading but located in regions with relatively cold‐microclimates and (2) understand whether there are distinct diatom responses to biotic nutrient loading (seabirds) and abiotic (climate) drivers of environmental change across a small geographical region. We predicted no synchronous changes between the diatom assemblages and seabird proxy (sediment ẟ^15^N) as previous research suggests climate is an overriding factor on Arctic pond biology (Griffiths et al., 2017; Keatley et al., 2008). Nonetheless, we predicted that diatoms will respond to climate change in the study ponds as they have done throughout the circumpolar Arctic (Kahlert et al., 2022; Smol et al., 2005); however, this response may be variable given the differences in pond size, depths, and microclimates across islands, leading to different lengths in pond ice cover and habitat variability. Furthermore, as these are shallow ponds with significant moss development around their shorelines, we predict that the diatom response to climate change may be recorded by epiphytic taxa.

## 2 METHODS

### 2.1 Site description

**FIGURE 1:**
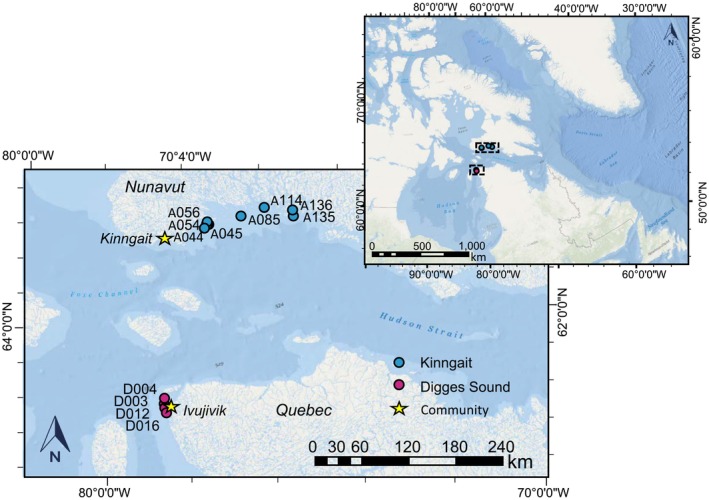
Map showing the location of islands with study ponds within the Hudson Strait region of the eastern Canadian sub‐Arctic. The closest communities to the study ponds are Kinngait (formally Cape Dorset), Nunavut, and Ivujivik, Québec.

Kinngait (previously known as Cape Dorset, Nunavut, Canada) and Ivujivik (Québec, Canada) are remote communities in the westerly arm of the Hudson Strait (Figure 1). Both areas have several nearby small, uninhabited islands on which seabirds, predominantly northern Common Eiders (*Somateria mollissima borealis*), nest. The two study regions exhibit similar geography and topography, both categorized as the Meta Incognita ecoregion (Sanborn‐Barrie et al., 2008). The area is characterized by continuous permafrost and rugged bedrock, with minor amounts of colluvial soils (3vGeomatics Inc., 2011). Natural vegetation is dwarfed due to high winds, frigid temperatures, and poor soils (Ricketts et al., 1999). Nutrient‐rich guano from the nesting seaducks visibly promotes the growth of catchment vegetation (Clyde et al., 2021; Duda et al., 2018). Sampling locations were given unofficial names by the monitoring program at Environment Canada and Climate Change (ECCC; Table 1). Previous analysis on pond water chemistry across these same islands confirmed seaducks have an impact on pond water chemistry with pond water from seaduck‐affected sites having elevated metal (Al, Cd, and Zn), metalloid (Se), and nutrient concentrations (N and P; Duda et al., 2018). Moreover, δ^15^N and sterol and stanols measured in the same cores established the long‐term presence of seaducks in the region (Hargan et al., 2019).

**TABLE 1:** Locations of the 12 study ponds, with corresponding mean sediment core stable nitrogen isotope values (ẟ^15^N; ‰) and mean core C:N.

Pond	Official island name	Latitude (N)	Longitude (W)	Mean core ẟ^15^N (min to max range)	Mean core C:N (min to max range)
A045	Tunitjuak Island	64°17′08″	75°46′56″	7.2 (6.3–8.5)	11.0 (8.7–13.0)
A054	Putaguk Island	64°19′04″	75°45′17″	10.2 (8.2–14.6)	9.8 (8.7–10.8)
A056	Qasigijjat	64°16′59″	75°44′25″	7.3 (6.0–8.8)	10.3 (8.6–11.5)
A114	Salunnaqtuuq	64°16′06″	74°11′20″	9.6 (9.2–10.5)	12.1 (11.4–13.0)
D003	North Skerries	62°25′52″	78°10′17″	7.8 (7.4–8.3)	11.5 (8.6–13.0)
D004	North Skerries	62°26′37″	78°09′02″	7.7 (surface sediment)	9.0 (surface sediment)
D012	South Skerries	62°23′01″	78°11′06″	8.9 (7.7–9.3)	8.7 (8.1–9.9)
A044	Simikutak	64°17′52″	75°47′14″	13.9 (12.4–15.7)	10.0 (9.3–10.6)
A085	Inugiavvik	64°17′32″	74°38′56″	11.6 (surface sediment)	8.5 (8.0–9.4)
A135	Qujjautaq	64°03′44″	73°31′56″	11.5 (10.5–13.7)	10.9 (9.5–12.6)
A136	N/A	64°05′07″	73°30′44″	10.0 (9.3–13.6)	9.5 (8.4–10.2)
D016	Île Pikiulik	62°19′20″	78°10′33″	10.3 (9.8–11.0)	10.9 (10.6–11.4)

### 2.2 Climate data

Climate data from the municipalities of Ivujivik and Kinngait were obtained from the Climate Atlas of Canada (Prairie Climate Centre 2019). The source of the historical data used in the atlas is Natural Resources Canada (NRCan; McKenney et al., 2011). This dataset is comprised of daily mean, maximum, and minimum air temperature, and daily precipitation totals for the same 10 km by 10 km grid used in the downscaled global climate model datasets produced by Pacific Climate Impacts Consortium. The variables examined in relation to the sedimentary data are mean annual temperature (°C), mean annual precipitation (mm), and degree days greater than 5°C.

### 2.3 Sample collection

Sediment core collection took place over two field seasons in two coastal locations in the Hudson Strait region of northern Canada. A total of 12 sediment cores were collected from seaduck colonies visited near Ivujivik, Québec in July 2014, and near Kinngait, Nunavut in July 2015 (Table 1). From Ivujivik, islands were accessed southwest of the community within Digges Sound (62°33′N, 77°35′W), located at the northeastern corner of Hudson Bay at the entrance of Hudson Strait. From Kinngait, islands were visited up to ~160 km southeast of the community along southern Baffin Island within Andrew Gordon Bay of Foxe Peninsula. Islands were chosen based on previous eider colony surveys, to attempt to sample islands that supported nesting seaducks (>200 nests; Duda et al., 2018; Iverson et al., 2014). A sediment core was collected from the center of the main pond on each island using a push corer (Glew & Smol, 2016), and core intervals were extruded on site at 0.5 cm resolution to the base of the sediment core using a Glew (1988) vertical extruder. Sediment cores were frozen at the University of Ottawa until sub‐sampling for dating and each proxy. As diatoms respond sensitively to changes in pH, we measured the acidity of eider guano to holistically discuss the effect of seaducks on the diatom community. We measured the acidity of three Common Eider fecal samples collected during fieldwork. Each sample was measured in triplicate using a Hannah pHEP meter in a consistent 1:10 sample to deionized water mass ratio, as recommended for highly organic material (Hendershot et al., 2008).

### 2.4 Sediment core dating and age chronologies

Chronologies for most of the sediment cores have previously been published in Hargan et al. (2019). Briefly, a chronology was determined for each core using a Ortec High Purity Germanium Gamma Spectrometer (Oak Ridge, TN, USA) to measure the activity of radionuclides (e.g., ^210^Pb, ^214^Pb, and ^137^Cs; Appleby, 2001; Schelske et al., 1994). Certified reference materials obtained from the International Atomic Energy Association (Vienna, Austria) were used for efficiency corrections, and chronologies were developed using ScienTissiME (Barry's Bay, ON, Canada). All sediment chronologies were calculated using the constant rate of supply (CRS) model (Appleby, 2001). Second order polynomial equations were fitted through the CRS dates to infer dates beyond the range of ^210^Pb activity (published in Hargan et al., 2019). Since Hargan et al. (2019), three additional cores (A136, A085, and D004) have been dated.

### 2.5 Diatoms

Sediment samples were prepared for diatom analyses following standard procedures outlined in Wilson et al. (1996). Briefly, ~0.5 g of wet sediment was treated with a 1:1 molar ratio of concentrated sulfuric (H_2_SO_4_) and nitric (HNO_3_) acids to digest the organic content of the sediment and isolate the siliceous material. The samples were placed in an 80°C water bath for ~3 h to digest the organic matrix. Samples were rinsed with deionized water daily until they reached a neutral pH. Slurries were pipetted onto cover slips, dried, and mounted on glass microscope slides using Naphrax®. A minimum of 400 diatom valves were counted for each sedimentary interval using a Leica DMRB light microscope fitted with differential interference contrast optics at 1000× magnification. Diatoms were identified to the lowest taxonomic level possible using a selection of taxonomic references (Antoniades et al., 2008; Krammer & Lange‐Bertalot, 1986–1991).

### 2.6 Sedimentary chlorophyll *a*

Visible range spectroscopy (VRS) was used to infer concentrations of sedimentary chlorophyll *a* (chl *a*), which is a proxy for whole‐lake primary production (Michelutti, Blais, Cumming, et al., 2010; Michelutti, Blais, Mallory, et al., 2010; Wolfe et al., 2006). VRS methods measure the entire suite of chl *a* degradation products (Michelutti, Blais, Cumming, et al., 2010; Michelutti & Smol, 2016; Wolfe et al., 2006). In preparation for spectral analysis, sediments were freeze‐dried, sieved through a 125 μm mesh, with sediment ~2–3 mm in size placed into a 19 × 65 mm glass vial inside a FOSS NIRSystem Model 6500 rapid content analyzer. Chl *a* was measured for all cores except for D004, which was a short core, encompassing <100 years (as discussed below). A simple metric (the area under the absorbance peak from 650 to 700 nm) was used to infer chl *a* concentrations using log transformed data from Michelutti, Blais, Cumming, et al. (2010) and Michelutti, Blais, Mallory, et al. (2010) with the equation:

$$\begin{array}{c} \text{Chlorophyll}\;a+\text{derivatives}=\\ \;\exp\left(0.83784^{*}\ln\left(\text{peak}\;\text{area}\;650-700\;\mathrm{nm}\right)+\left(-2.48861\right)\right). \end{array}$$

### 2.7 Elemental and stable nitrogen isotope analyses

Sediment samples were analyzed for stable isotopes of nitrogen, as well as for %C and %N, at the Ján Veizer Stable Isotope Laboratory (University of Ottawa, ON). The molar ratio of C:N can be used as an approximate measure to assess whether sedimentary organic matter is largely of aquatic (C:N < 10) or terrestrial (C:N > 20) origin (Meyers & Ishiwatari, 1993). For elemental %C and %N analysis, sediment samples and standards were analyzed using a Vario EL III Elemental Analyzer (Elementar, Germany). Sediment amounts needed for the δ^15^N isotopic analyses were determined based on the results of the elemental analysis and weighed accordingly into tin capsules with two parts tungsten trioxide (WO_3_). The isotopic composition of nitrogen was determined by the analysis of N_2_, produced by combustion on a VarioEL III Elemental Analyzer (Elementar, Germany) followed by “trap and purge” separation and online analysis by continuous‐flow with a DeltaPlus XP Plus Advantage Isotope Ratio Mass Spectrometer coupled with a ConFlo II (Thermo, Germany). Our δ^15^N data were reported using delta (δ) notation in parts per thousand (‰) enrichments or depletions relative to common standards (AIR for δ^15^N). Isotope data were normalized using previously calibrated internal standards, and analytical precision was ±0.2%. δ^15^N data was not obtained for A085 and C:N data for D004, D016, and A085.

### 2.8 Numerical analysis

Diatom assemblage data are reported as relative abundances. Common taxa from each core were plotted in a stratigraphy, which we define as >10% relative abundance and present in at least one interval. To explore whether diatom species data, summarized as species diversity (Hill's N2; Hill, 1973), followed similar patterns to that of seabird‐derived nutrients (as estimated by δ^15^N values) or to trends in whole‐lake primary production (as estimated by sedimentary chl *a*), we used parametric Pearson correlations between these summary indicators. Hill's N2, C:N, and δ^15^N values were fitted using generalized additive models (GAMs) using the methods suggested by Simpson (2018) to account for heteroscedasticity in paleolimnological data. Periods of statistically significant change were determined by identifying periods of nonzero rate of change in the GAMs first derivative (Simpson, 2018). These statistical analyses were completed using the packages mgcv v.1.8‐31 (Wood, 2017) and gratia v.0.6.0 (Simpson, 2021) in the R workspace (R Core Team, 2012).

## 3 RESULTS

### 3.1 Climate

**FIGURE 2:**
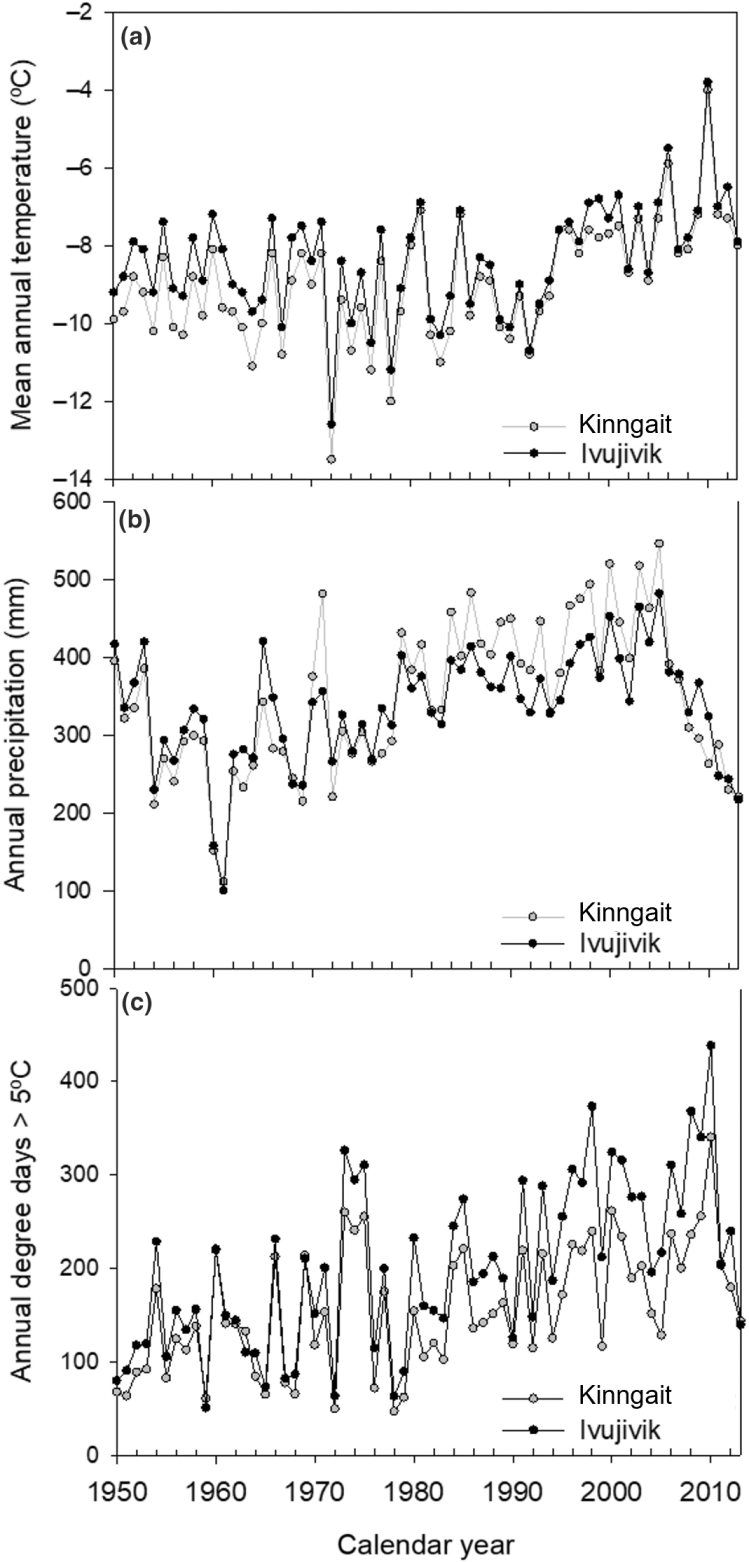
Mean annual air temperature, precipitation, and degree days >5°C for Kinngait, NU (previously Cape Dorset) and Ivujivik, QC regions from 1950 to 2013 (Data source: Climate Atlas of Canada; Prairie Climate Centre 2019, and Natural Resources Canada; McKenney et al., 2011).

The available climate data from Kinngait and Ivujivik appear to have coherent trends in mean annual temperature, annual precipitation, and annual days >5°C (Figure 2). From 1950 to 2013, the mean annual temperature in the study region was −8.4°C, annual precipitation was 337 mm, and annual days >5°C was 200.5 (McKenney et al., 2011; Figure 2). From our limited temporal record, it appears that the number of annual days >5°C has been increasing.

### 3.2 Sediment core chronologies

**FIGURE 3:**
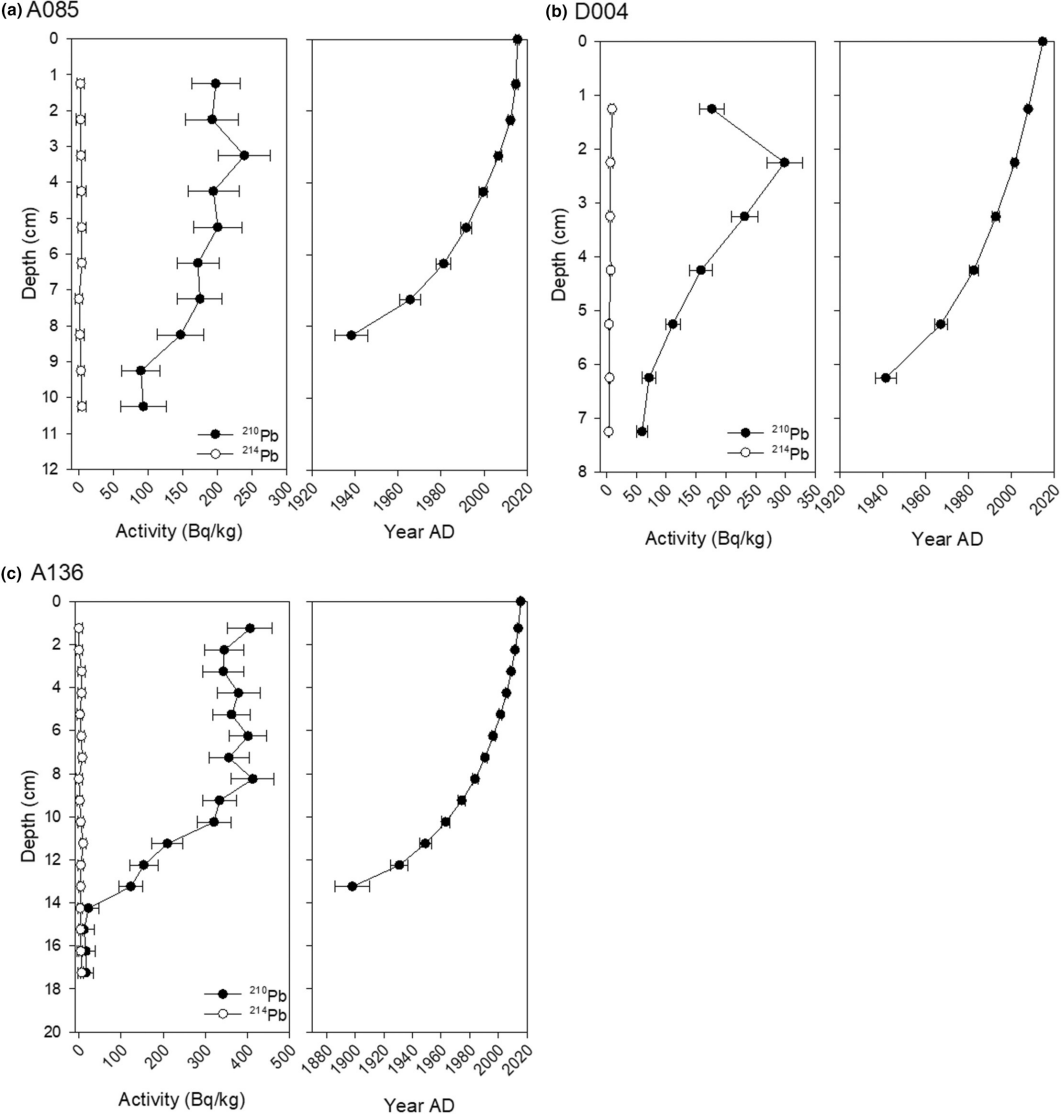
^210^Pb activities and constant rate of supply (CRS) ^210^Pb‐chronologies for sediment cores: (a) A085, (b) D004, and (c) A136.

Profiles of the ^210^Pb activities for many of the sediment cores analyzed in this paper have previously been published in Hargan et al. (2019). To briefly summarize, excess ^210^Pb profiles for A135, A044, and A056 followed a classic exponential decline through depth, with highest initial ^210^Pb activity levels measured in A044. Alternatively, several of the sediment cores showed greater fluctuation in initial ^210^Pb activity levels with some plateaus in the first few centimeters, but all the cores still exhibited a clear ^210^Pb decay. Sediment cores A045, A056, and A054 have distinct peaks in ^137^Cs that align well with ~1963, as inferred by CRS models. Extrapolation of the resulting ^210^Pb chronologies indicates that the Kinngait cores collected in 2015 capture longer time periods, ~160 to 400 years before present, compared with the Digges Sound cores collected in 2014 which capture between 120 and 210 years before present. The polynomial equations used to extrapolate the CRS dates beyond the range of ^210^Pb all display strong correlations, *R* ^2^ values >.97. In this paper, we publish new sedimentary chronologies for A136, A085, and D004 (Figure 3). D004 is a short core of 7 cm and captures ~65 years of sedimentation. A085 also encompasses a relatively short time span relative to the other eider cores collected from islands near Kinngait. Here, the last ^210^Pb CRS date is ~1930 CE at 8 cm (of 12 cm core). A136 has relatively stable ^210^Pb activities from 0 to 8 cm and at ~8 cm ^210^Pb begins decaying and reaches background ^214^Pb activities at ~14 cm (Figure 3).

### 3.3 Diatom assemblages

**FIGURE 4:**
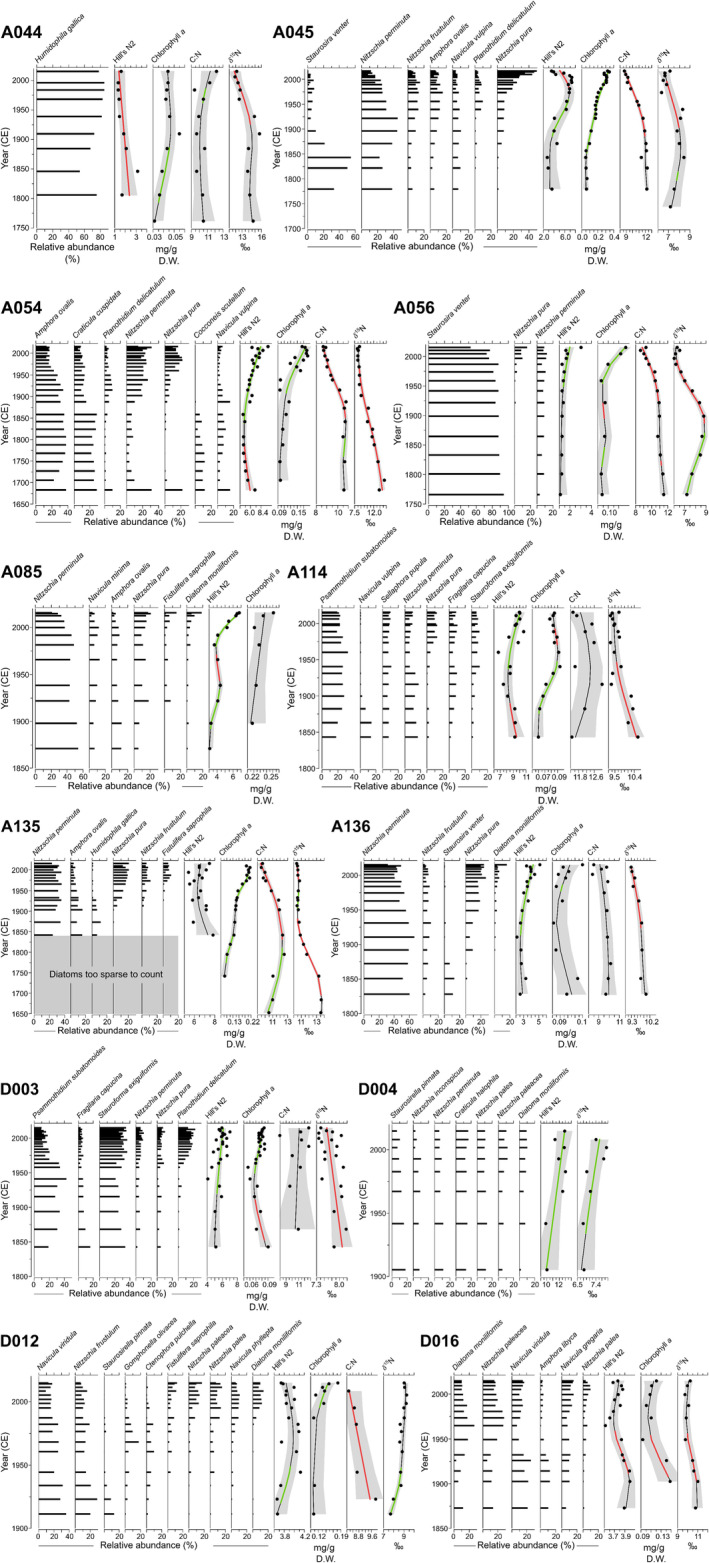
Changes in diatom assemblages, sedimentary chlorophyll *a* concentrations, Hill's N2, C:N, and δ^15^N values for each study pond. The illustrated taxa were in >10% relative abundance in at least one interval. Hill's N2, C:N, and δ^15^N values are fitted using generalized additive models (GAMs). The shaded bands are the 95% pointwise confidence intervals. Significant periods of increase and decrease are bolded in green and red, respectively.

The sedimentary diatom assemblages from all 12 ponds in this study recorded several changes over the last ~200 years (Figure 4). Additionally, given the broad geographic distribution of these islands (<1 km to ~300 km between islands), several diatom taxa co‐occurred across the sediment cores and we observed similar changes in diatom assemblage composition through time at many ponds. Common co‐occurring diatom taxa in these eider pond sediment cores included many periphytic *Nitzschia* species (*Nitzschia pura*, *N*. *perminuta*, *N*. *palea*, and *N*. *frustulum*), *Achnanthidium minutissimum*, *A*. *delicatula*, *Diatoma moniliformis*, *Staurosira venter*, *Fragilaria capucina*, *Psammothidium subatomoides*, *Navicula vulpina*, and *Amphora ovalis* (Figure 4). The most prevalent diatom assemblage change observed across all cores was the increase in the percent relative abundance of *Nitzschia pura* from older to modern sediments. This increase in *N*. *pura* ranges from a minimum of 3% in D004 to a maximum relative abundance change of 21% in A054, 16% in A056, 49% in A045, and 21% in A135 (Figure 4). However, many ponds also demonstrated consecutive increases in other *Nitzchia* taxa along with *N*. *pura*. For example, pond D012 captured a consecutive increase in *Nitzschia palea*, *N*. *paleacea*, and *N*. *pusilla* and pond D016 in *N*. *palea* and *N*. *paleacea*. The diatom *Nitzschia perminuta* shows variable changes in percent relative abundance from older to modern sediments across the ponds. In many cases, this epiphytic moss taxon was present at the base of the sediment cores (A136, A056, A085, A054, A045, and A135), corroborating the presence of marine‐derived nutrients entering the ponds for several centuries. Diatom taxa at the base of sediment cores were more variable across ponds compared with modern assemblages, and we observed declines in the percent relative abundance of different taxa across ponds including *S*. *venter*, *N*. *vulpina*, *Cocconeis scutellum*, and *Amphora* species (Figure 4). Several sediment cores are also marked by the presence of taxa with distinct habitat optima including hyper‐eutrophic diatom *Fistulifera saprophila* which is present in A135, A085, and D012, and aerophilic *Humidophila gallica*, the dominant species in pond A044.

Diatom diversity was variable across the study ponds; however, the prevailing trend suggested increasing diversity (as determined using Hill's N2) in correspondence with the period of warming from ca. 1900 until present. Of the 12 study ponds, 58% increased during this period (A054, A056, A085, A114, A136, D003, and D004), 17% declined (A044 and A045), and 25% were unchanging (A135, D012, and D016; Figure 4).

### 3.4 Sedimentary chlorophyll *a*

Across most of the sediment cores, there was a general increasing trend in chl *a* through time, with the largest increase in sedimentary chl *a* recorded in pond A045 (Figure 4). D016 and A054 sediment cores track early initial declines in chl *a*, before increases again until the surface. The chl *a* increases in many sediment cores, including A135, A056, A054, D012, D016, and A085, are synchronous in timing with the main break in the diatom assemblage data (Figure 4).

### 3.5 C:N ratios & δ^15^N values

Sedimentary organic matter is largely of aquatic origin with most ratios of C:N hovering around 10, and all ratios were <20 (Table 1). Ponds A135, A056, A054, and A045 captured significant declining C:N over the majority of the sediment record (Figure 4). Trends in ẟ^15^N values across cores are summarized in Hargan et al. (2019). Eider pond sediment profiles showed enriched δ^15^N values (relative to Hudson Strait sediment cores with no marine bird colonies within their catchments) from the base of the cores to the present (Figure 4). Generally, many of the Cape Dorset records show declines in ẟ^15^N values in the early to mid‐20th century. The two longest records representing ~350 years (A135 and A054) show a continuous gradual decline in δ^15^N values from ~1620 AD to present (−3.2‰, −6.2‰ respectively), whereas the historical sedimentary δ^15^N values for three islands (A044, A045, and A056) peaked at the turn of the 20th century until ~1930 AD and then declined to the present (−3.8‰, −2.1‰, and −2.8‰ respectively; Figure 4). Fluctuations in δ^15^N values in the sedimentary records from Digges Sound are less variable than the islands supporting larger eider colonies southwest of Baffin Island. D003 has stable δ^15^N values from ~1800 to present (7.8 ± 0.26‰, greatest change −0.86‰), and the δ^15^N values of D012 gradually increased from the base of the recorded from 1900 to 2015 (8.8 ± 0.43‰; Figure 4). From ~1950 to 2000, the δ^15^N values for D016 are on average 1‰ lower than from ~1870–1926.

### 3.6 pH measurements of Common Eider feces

The pH of Common Eider feces was measured from three birds in triplicate. Feces was alkaline, with a pH of 8.1 ± 0.99. The alkaline feces, caused by the eider's molluscivorous feeding behavior, are likely important factors affecting the water chemistry of ponds impacted by the seaducks.

### 3.7 Statistical comparisons between proxies

Statistically significant correlations between proxies were variable across ponds. There is a significant, negative correlation between chl *a* and δ^15^N in 5 of 11 ponds, and a significant, positive correlation to δ^15^N values in one pond. Significant, positive correlations between chl *a* and diatom diversity (Hill's N2) occur in 6 of 11 sediment cores (Table 2). Although sedimentary C:N ratios were only measured in few sediment cores, it appears that there are consistent negative correlations between C:N ratios and chl *a*.

**TABLE 2:** Pearson correlations between the various paleo‐indicators (chlorophyll *a*, ẟ^15^N values, Hills N2, C:N ratios) from each eider pond.

Pond		N2	ẟ^15^N	C:N
A054	N2			
	ẟ^15^N	**.809**		
	C:N	−**.776**		
	Chl *a*	**.733**	**−.737**	**−.805**
A056	N2			
	ẟ^15^N	**−**.492		
	C:N	**−.604**	**.761**	
	Chl *a*	**.802**	**−.628**	**−.895**
A135	N2			
	ẟ^15^N	−.126		
	C:N	.140	.060	
	Chl *a*	−.254	**−.671**	**−.853**
A136	N2			
	ẟ^15^N			
	C:N	**−.805**		
	Chl *a*	**.532**	.632	−.395
A044	N2			
	ẟ^15^N	.305		
	C:N	−.271	−.540	
	Chl *a*	−.239	.128	**−**.105
A045	N2			
	ẟ^15^N	**−.684**		
	C:N	**−.601**	**.631**	
	Chl *a*	**.571**	**−.716**	**−.955**
A114	N2			
	ẟ^15^N	−.031		
	C:N	−.374	−.326	
	Chl *a*	−.097	**−.722**	.063
A085	N2			
	Chl *a*	**.793**		
D003	N2			
	ẟ^15^N	−.110		
	C:N	.051	−.500	
	Chl *a*	.215	−.191	.098
D012	N2			
	ẟ^15^N	.365		
	Chl *a*	−.029	.353	
D016	N2			
	ẟ^15^N	**.694**		
	Chl *a*	**.644**	**.924**	

*Note*: Significant correlations (*p* ≤ .05) are indicated in bold. (Pond D004, a short core, was excluded because chl *a* and ẟ^15^N was not run).

## 4 DISCUSSION

### 4.1 Effects of seaducks on the environment

As with most seabirds that form large nesting colonies, Common Eiders act as ecosystem engineers by delivering marine‐derived nutrients that promote the growth of plants and formation of soils on otherwise nutrient‐poor islands (Clyde, 2016; Clyde et al., 2021; Duda, Glew, et al., 2020; Ebert et al., 2013; Vestbo et al., 2019). Enriched δ^15^N values from the base of all sediment cores to present indicate the eiders have been nesting on the islands for the time represented by the sedimentary records, ranging between ~115 and ~400 years old (Hargan et al., 2019). These marine‐derived seabird nutrients have allowed for the proliferation of mosses in and around the ponds and resulted in primarily epiphytic diatom assemblages.

Epiphytic diatom taxa are present to the base of sediment records, including *Nitzschia perminuta*, *N*. *pura*, *Diatoma moniliformis*, and *Amphora* species. *Amphora* species can have relatively high optima for key nutrients such as phosphorus (e.g., *Amphora pediculus* TP optima of 114.3 μg/L; Bennion, 1994) and *A*. *ovalis* thrives during periods of nutrient enrichment including increasing with shifts in ẟ^15^N values and %N from Thule whaling (Hadley, Douglas, Blais, & Smol, 2010). Typically, before anthropogenic climate warming, sub‐Arctic and Arctic ponds are characterized by depauperate diatom assemblages of benthic epilithic taxa (Griffiths et al., 2017; Michelutti, McCleary, Antonaides, et al., 2013; Smol et al., 2005). Here, we recorded high diatom diversity (Figure 4) to the base of several sediment cores, which can be linked to the historical seabird nutrients that result in greater habitat complexity (e.g., moss substrates) compared with oligotrophic ponds that are typically dominated by rocky substrates (e.g., reference ponds on southern Baffin Island near Kimmirut in Michelutti, McCleary, Antonaides, et al., 2013). *Stauroneis venter* is present in several eider ponds to the base of the cores (A045 and A056), and we hypothesize this taxon occurs in the deeper ponds where rocky substrate remains at deeper depths, and prolonged ice cover into the summer season may limit epiphytic taxa from completely dominating the assemblage.

As part of the seaduck‐island engineering process, greater soil accumulation around pond edges (Clyde, 2016; Clyde et al., 2021) increases pond depth, supporting the formation of more permanent ponds when some ponds may have historically been ephemeral by late summer. Changes in the relative abundance of aerophilic diatom taxa in ponds A044 and A135 indicate that ponds have become more permanent features on these islands. For example, we tracked a decline in aerophilic diatom, *Humidophila gallica*, from the base of A135 until ~1925. The sediment record of A044 is still dominated by *H*. *gallica*, suggesting that this pond remains ephemeral to 2015 when the core was collected. This pond is perched at the highest point on an island and likely receives less catchment drainage from snow melt. Most aerophilic diatom species are indicative of low nutrient availability. Of the 122 diatoms listed as exclusively aerial or occurring in mostly wet, moist, or temporarily dry places in Europe, van Dam et al. (1994) considered only four of the taxa to be facultatively or obligately nitrogen‐heterotrophic taxa, while over half of those characterized with regard to nitrogen uptake were classified as tolerating only very small concentrations of organic nitrogen (Johansen, 2010). This indicates that early in the history of these sub‐Arctic island ponds, when they were likely seasonally ephemeral, there would have been few diatom taxa capable of withstanding the high organic nitrogen loads from birds and the dry conditions.

### 4.2 Variability in the region

While diatoms may track seabird population dynamics when there are large shifts in ẟ^15^N values, including in ponds in the High Arctic (Keatley et al., 2011), this was not consistent across all our sub‐Arctic study ponds. The establishment of eider colonies hundreds of years ago introduced substantial nutrients to catchments and ponds. These nutrients likely remain circulating within island ecosystems for several decades, even when bird populations decline or are absent. Clyde (2016) observed deep, nutrient‐rich soils on eider islands compared with no colony islands and mainland sites. While we observed large declines in sediment core ẟ^15^N values, often on the order of ~3‰, we inferred this to indicate declining nesting eider populations (Hargan et al., 2019), we do not fully anticipate that this change in ẟ^15^N values and respective changes in %N would cause a substantial response in diatom assemblages. Here, our evidence suggests that the high and relatively continuous pulse of nutrients (compared with Arctic ponds with no seabirds present) structured the initial diatom assemblages as moss substrates appeared in these ponds. Over time, however, declines in the delivery of nutrients to islands are not registered well with diatoms and pond primary production due to a sustained nutrient reservoir and moss habitat, and the overriding influence of climate in Arctic regions. The slow breakdown of nutrients in Arctic regions has been widely observed at Thule sites on both Ellesmere and Baffin Islands, where whalebone houses that were constructed >1000 years ago remain to represent a significant input of nutrients to ponds (Hadley et al. 2010). On Baffin Island near Kimmirut, approximately ~250 km east from A135, slow rates of decomposition of butchered marine animal bones also continue to introduce nutrients to the freshwater sites into which they drain (Michelutti, McCleary, Antonaides, et al., 2013). There are notable, albeit rare taxa with high nutrient optima, which are consistent with biotic nutrient introductions, such as *Fistulifera saprophila* in D012, A135, and A085 (Figure 4). This taxon was found in Resolute sewage ponds (Stewart et al., 2014) and Annak Lake on Belcher Islands, also with high nutrient concentrations from sewage inputs (Michelutti et al., 2007).

Frequently, nutrient loading by certain seabirds results in an acidification of lakes and ponds (Duda, Allen‐Mahé, et al., 2020; Duda, Michelutti, et al., 2021; Duda, Robertson, et al., 2020; Gonzalez‐Bergonzoni et al., 2017) as the main component of guano is uric acid. As diatoms often respond very sensitively to pH changes (Battarbee et al., 2001), diatoms in avian‐acidified lakes closely track seabird population dynamics (Duda, Allen‐Mahé, et al., 2020, Duda, Robertson, et al., 2020). For example, on Baccalieu Island in Newfoundland, not only do Leach's Storm‐Petrels have acidic guano, but they burrow, introducing organic carbon to water and acidifying the water further (Duda, Allen‐Mahé, et al., 2020, Duda, Robertson, et al., 2020). In our Arctic ponds, we observed alkaline pH waters (8.5–10). Eiders are molluscivorous, producing guano with an alkaline pH of 8.1 ± 0.99, as well as introducing calcium to ponds via their feeding behavior (Ebert et al., 2013). The high pond pH likely occurs both because of alkaline eider guano and because of enhanced aquatic production stimulated by the nutrients from seabird wastes, combined with 24‐h daylight that does not limit photosynthesis (Keatley et al., 2009; Stewart et al., 2013). These seasonal physical conditions in the Arctic appear to buffer pH acidity from seabirds such as Northern Fulmars (Keatley et al., 2009), eiders, terns (Michelutti, Blais, Cumming, et al., 2010; Michelutti, Blais, Mallory, et al., 2010), and geese (Jensen et al., 2019; MacDonald et al., 2014) as well as pond anoxia, which occurs in temperate ponds experiencing greater diurnal cycles of photorespiration, although temperate shallow rock pools influenced by gull guano consistently exceeded a pH of 10, which was linked to high photosynthetic activity (Loder et al., 1996). High alkalinity in Arctic ponds impacted by birds is not uncommon. For example, Arctic ponds disturbed by geese recorded pH ranges from 7.4 to 9.4 (Jensen et al., 2019). Likewise, geese‐affected ponds near Churchill (Manitoba) recorded increases in pH through the open‐water season due to increasing aquatic productivity and chemically enhanced CO_2_ invasion (MacDonald et al., 2014, 2015). Furthermore, ponds in the High Arctic impacted by nesting fulmars had highly alkaline waters compared with local ponds not receiving seabird nutrients (Keatley et al., 2009). Although we did not measure oxygen levels to support that heightened photosynthesis supersedes decomposition, Jensen et al. (2019) found high oxygen saturation >80% in geese‐impacted ponds and concluded that strong oxygen depletion is unlikely in shallow, wind‐mixed Arctic ponds.

### 4.3 Climate as a co‐factor

Many of the changes recorded in the diatom community and chl *a*, although variable, are most likely driven by climate change. Most notably, recorded changes in chl *a* are not linked to changing seabird population dynamics as we found significant, negative correlations between chl *a* and ẟ^15^N values in 5 of 11 cores, and no correlations in the other cores (Table 2). On Digges Island, adjacent to the eider islands in Digges Sound, a large colony of thick‐billed murres (~1 million birds) introduces nutrients to nearby lakes as evidenced by elevated ẟ^15^N values relative to reference lakes; however, diatom assemblages were indistinguishable between the impact and reference lakes (Hargan et al., 2017). Furthermore, monitoring of the thick‐billed murre colony over the last few decades indicates population size has not changed substantially (Gaston, 2003) and yet, 20th century changes in diatom assemblages occur across all lakes consistent with warming (Hargan et al., 2017). Many of the diatom changes are consistent with longer ice‐free conditions and growing seasons. Declines in the relative abundances of *Stauroneis venter* (A045, A056, and A136) and benthic epilithic *Navicula vulpina* and *N*. *viridula* in several ponds generally indicate longer growing seasons as more complex substrates develop in ponds, allowing epiphytic taxa to flourish. During cooler periods, the persistence of ice cover can reduce primary production by limiting light penetration into the pond. Under warmer conditions, ice cover is reduced, more light is available for photosynthesis during longer growing seasons, and as a result, more habitat becomes available for colonization by algae (Rühland et al., 2015; Smol, 2023). Although temperature records are short for our study region, it is evident that the number of degree days >5°C has slowly been increasing since 1950 with a jump in the 1980s and that there were substantial increases in mean annual air temperatures beginning in the 1990s and declines in precipitation in the early 2000s (Figure 4). Several of the ponds demonstrated diatom assemblage changes, increased diatom diversity, and chl *a* changes beginning in the late‐19th century, when many Arctic ponds demonstrated climate‐related changes (Rühland et al., 2008), as well as changes in the same proxy data in the 1970s (A045, A135, A054, and D003) and the 1990s (A085, A056, and D012), in line with more severe contemporary climate warming.

Variability in the timing of pond diatom assemblage changes linked to climate warming is to be expected, given differences in lake morphometry and microclimate across sites. For example, two lakes on Ellesmere Island exhibited strikingly different paleolimnological diatom profiles, despite their physical proximity, similar depths, and nearly identical water chemistry (Keatley et al., 2008). In that study, the differential diatom response was linked to the effects of local shading by hills, which yielded different microclimates for ponds which were only a few meters apart. In turn, these different microclimates alter lake ice cover dynamics and thus become the principal determinant of shifting diatom assemblages (Keatley et al., 2008). Additionally, diatom changes within a diatom functional group, such as between epiphytic taxa, are more challenging to interpret compared with changes in the relative abundance of diatoms between functional groups/guilds. In many of the cores, we observe changes between *Nitzschia* species, for which we have limited autecological knowledge.

## 5 CONCLUSIONS

Diatom assemblages in Arctic ponds capture a nutrient signal from nesting seabirds as evidenced by the diverse epiphytic diatom assemblages to the base of sediment records; however, the diatom assemblages appear less sensitive to changes in nutrient pulses over time compared with temperate regions. Here, the sub‐Arctic diatom response to seabirds is mainly due to changes in habitat change, such as the growth of moss over the pond's rocky substrate. Once mosses were established at the site, we did not observe responses of diatom assemblages to declines in seabird nutrients. The sites are all shallow ponds, and if planktonic taxa were present, we may well have seen greater sensitivity to direct water chemistry, which reflects divergent ecophysiologies between benthic and planktonic algal communities in high latitude waterbodies. Similarly, the diatom response to anthropogenic climate warming occurs within the epiphytic diatom community and is muted relative to other Arctic shallow ponds where a striking switch between epilithic to epiphytic diatom taxa has been observed. However, the ponds do appear to be responding to climate warming with changes in epiphytic taxa aligning with increases in chlorophyll *a* and diatom diversity.

Overall, using diatom assemblages to interpret changes in seabird populations is complicated in Arctic regions, especially in shallow ponds where we observe indirect responses to seabird nutrients through changes in habitat and between epiphytic taxa inhabiting mosses. Certainly, in sediment cores, diatoms would be reliable at tracking the arrival and establishment of Arctic seabird colonies, but less so at changes in seabird population size. We suggest that diatom assemblage changes should not be used as the sole proxy to track bird population changes in Arctic regions. Rather, biogeochemical proxies like stable nitrogen isotopes and fecal sterols and stanols that originate directly from seabirds more reliably track historical population dynamics.

## AUTHOR CONTRIBUTIONS

**Kathryn E. Hargan:** Conceptualization (equal); data curation (lead); formal analysis (lead); funding acquisition (equal); investigation (equal); methodology (equal); validation (equal); visualization (equal); writing – original draft (lead). **Matthew P. Duda:** Data curation (supporting); formal analysis (equal); investigation (supporting); visualization (supporting); writing – review and editing (supporting). **Neal Michelutti:** Data curation (supporting); investigation (supporting); validation (supporting); writing – review and editing (supporting). **Jules M. Blais:** Conceptualization (supporting); funding acquisition (lead); investigation (supporting); methodology (equal); project administration (supporting); resources (equal); supervision (equal); writing – review and editing (supporting). **John P. Smol:** Conceptualization (equal); funding acquisition (equal); methodology (equal); project administration (equal); resources (equal); supervision (supporting); validation (supporting); writing – review and editing (supporting).

## CONFLICT OF INTEREST STATEMENT

The authors have no conflicts of interest to declare.

## Data Availability

Data and code used in the analyses are available via the figshare online repository: https://figshare.com/s/6036192c54b6ab765942 & https://figshare.com/s/2b57d5892d45a29726d9.
